# Molecular Characterization and Expression Analysis of IL-10 and IL-6 in Channel Catfish (*Ictalurus punctatus*)

**DOI:** 10.3390/pathogens12070886

**Published:** 2023-06-29

**Authors:** Xia Zhu, Yongtao Liu, Ning Xu, Xiaohui Ai, Yibin Yang

**Affiliations:** Yangtze River Fisheries Research Institute, Chinese Academy of Fishery Sciences, Wuhan 430223, China; zhuxia@yfi.ac.cn (X.Z.);

**Keywords:** channel catfish, interleukin-10, interleukin-6, immune response

## Abstract

IL-10 and IL-6 play important roles in protecting against inflammation and clearing pathogens from the body. In this study, homologous compounds of IL-10 and IL-6 were identified in channel catfish, and their immune responses were analyzed. The CDS sequences of IL-10 and IL-6 were 549 bp and 642 bp, respectively, and showed the highest homology with *Ameiurus melas*. In addition, the expression of the IL-10 and IL-6 genes was ubiquitous in 10 tissues examined. IL-10 is highly expressed in the liver and slightly expressed in the gill. The high expression of the IL-6 gene was observed in the spleen, heart, and gonad, with the lowest levels in the liver. LPS, Poly(I:C), PHA, and PMA showed a highly significant increase in IL-10 and IL-6 expression 48 h after CCK stimulation (*p* < 0.01). Otherwise, Yersinia ruckeri, Streptococcus iniae, channel catfish virus, and deltamethrin induced IL-10 and IL-6 expression, varying in intensity between different organs. Our results suggest that IL-10 and IL-6 are involved in the immune response of the host against the pathogen.

## 1. Introduction

Cytokines are small proteins secreted by the host in response to infection, immune responses, and various trauma. The immune signaling pathways involved in the cytokines include a variety of biological reactions in which interleukins, a group of cytokines, usually play an important role. Mammalian IL-10 is a pleiotropic cytokine that is considered as anti-inflammatory, while IL-6 is a pleiotropic cytokine with both pro-inflammatory and anti-inflammatory properties. Both IL-10 and IL-6 promote B cell differentiation and antibody secretion. The cytokines of the IL-10 family mainly include IL-10, IL-19, IL-20, IL-22, IL-24, and IL-26, which all share IL-10R2 [1]. The cytokines of the IL-10 family were identified before adaptive IL-10 was first discovered in 1989 by Mosmann [2] and were cloned from T-helper type 2 (Th2) cells. IL-10 often controls the inflammatory response by inhibiting the synthesis of various cytokines, including interferon-gamma, tumor necrosis factor, IL-1, IL-2, IL-3, IL-6, IL-8, and IL-12 [3]; hence, it was initially named as cytokine synthesis inhibitory factor (CSIF) [4] and was then renamed as IL-10.

At present, IL-10 has been extensively studied in several teleost fishes, such as golden pompano (*Trachinotus ovatus*) [5], Indian major carp (*Catla catla*) [6], grass carp (*Ctenopharyngodon idellus*) [7], zebrafish (*Danio rerio*) [8], and rainbow trout (*Oncorhynchus mykiss*) [9]. The production of IL-10 in fish may depend on the stimulation of immune processes in cells and immune organs. For example, IL-10 expression is known to be induced following pathogenic bacterial infection, as well as lipopolysaccharide (LPS) induction. IL-10 is a pleiotropic cytokine that can exert anti-inflammatory effects upon activation of the JAK/STAT signaling pathway.

IL-10 is an important anti-inflammatory and late cytokine that is produced following stimulation by pro-inflammatory mediators. In contrast, IL-6 is usually rapidly synthesized in response to infection by pathogens and tissue damage. IL-6 belongs to the IL-6 cytokine family and is a representative early inflammatory cytokine. It is generally accepted that classical signaling-mediated IL-6 exhibits regenerative or anti-inflammatory activities, whereas the pro-inflammatory responses of IL-6 are usually mediated by trans-signaling [10]. Initially, IL-6 was thought to be associated with antibody production in vertebrates due to its ability to induce the differentiation of B cells to plasma cells [11,12,13,14]. In mammals, IL-6 regulates immunoglobulin production, proliferation and the differentiation of lymphocytes, as well as inflammatory responses [15,16]. IL-6 plays a role both in the host response to tissue damage and infection and as a key factor in promoting inflammatory and autoimmune processes [17]. In fish, the IL-6 sequences of Japanese pufferfish (*Fugu rubripes*) [18] were reported in 2005, followed by rainbow trout [19], zebrafish [20], Siberian sturgeon (*Acipenser baeri*) [21], and *Megalobrama amblycephala* [22]. Interestingly, recombinant IL-6 rapidly increased the mRNA expression level of IL-10 in grass carp within a short period of time and was able to induce STAST phosphorylation in grass carp head kidney leukocytes [23]. Due to the important role played by IL-6 in fish, as demonstrated in the inflammatory response, lymphocyte differentiation [24,25], and the induction of microbial peptides [26]. Otherwise, the immunomodulatory effects of IL-6 was considered as immune enhancers, which can prevent disease or enhance treatment effects in fish. For example, the injected or oral administration of recombinant IL-6 after the attack of pathogenic microbes increased survival [21].

The channel catfish (*Ictalurus punctatus*) is native to the North American continent and gathers in southern Canada to northern Mexico [27]. At present, its domestic distribution is mainly concentrated in Hubei Province and the East China region and gradually forms an industrial development trend. Due to its high protein content and fast growth rate, channel catfish have become a popular aquaculture crop. However, outbreaks of diseases have restricted the sustainable development of the channel catfish breeding industry and annual productivity [27,28]. It is urgent to investigate the molecular mechanisms beyond the immune responses of channel catfish during pathogen infection. Based on this study, the cloning and sequence analysis of the IL-10 and IL-6 genes of the channel catfish and the response status of the channel catfish under different stimuli revealed their tissue distribution and response status under in vitro immune stimuli and further investigated their potential immunomodulatory effects. This study would deepen our understanding of the role of IL-10 and IL-6 in freshwater fish immunity.

## 2. Materials and Methods

### 2.1. Fish

Healthy, moderately sized (about 200 ± 20 g) channel catfish were purchased from the Baishazhou Aquatic Market, Wuhan Province, China, and were transported to the fish breeding base of the Yangtze River Fisheries Research Institute. All fish were kept in indoor circulating water tanks with freshwater at a water temperature of 25 ± 1 °C. The fish were given a two-week acclimatization period prior to treatment and were not fed during the experimental period.

### 2.2. Cloning of IL-10 and IL-6

The primers used in the experiments were designed based on the predicted sequences of IL-10 and IL-6 (GenBank accession number XM_017450800.1, XM_017455306.1, respectively) in channel catfish, and the primers are shown in Table 1. Total RNA was extracted from the spleen of the channel catfish using TRIzol reagent (Invitrogen, Carlsbad, CA, USA) according to the manufacturer’s instructions. The purity and concentration of total RNA were determined using a Nanodrop ND-2000 (Thermo Scientific, Waltham, MA, USA). The integrity of the total RNA was assessed using 1% agarose gel electrophoresis. First-strand cDNA was synthesized by taking 300 ng of total RNA using the reverse transcription kit (Yeasen Biotech, Shanghai, China) following the manufacturer’s instructions. The PCR reactions were performed in a total volume of 20 µL, including 10 µL EX Taq DNA polymerase (TaKaRa, Dalian, China), 1µL cDNA, 1µL of each primer, and 7 µL ddH_2_O. Then, the spleen cDNA was used as the template and amplified according to the following amplification procedure: 95 °C for 5 min; 95 °C for 30 s, 60 °C for 45 s, 72 °C for 80 s, and 72 °C for 15 min for 35 cycles. The PCR products were then sub-cloned into the pMD19-T vector (Takara, Dalian, China) and sequenced by a commercial company in Wuhan, China.

### 2.3. Bioinformatics Analysis of IL-10 and IL-6

The sequencing results were compared using Nucleotide BLAST: nucleotide databases were searched using a nucleotide query for sequence blast (https://blast.ncbi.nlm.nih.gov/Blast.cgi) (accessed on 1 May 2023). The open reading frame was found using the ORF Finder (http://www.ncbi.nlm.nih.gov/gorf/gorf.htmL) (accessed on 1 May 2023). The molecular mass and isoelectric point of the amino acid sequences was predicted using the ExPASy—ProtParam tool (http://expasy.org/tools/) (accessed on 1 May 2023). Protein multiple sequence alignment and beautification were performed using DNAMAN 9.0 and GeneDoc 2.7 software. The neighbor-joining (NJ) phylogenetic trees of the amino acid sequences of IL-10 and IL-6 were constructed using MEGA 6.0 software, respectively. The tFold-Server was used for the online structure prediction of the IL-10 and IL-6 proteins (https://drug.ai.tencent.com/console/cn/login?redirect=%2Fcn%2Ftfold) (accessed on 1 May 2023).

### 2.4. Tissue Expression Analysis of IL-10 and IL-6

To detect the expression of the IL-10 and IL-6 genes in 10 tissues of healthy channel catfish, including the hindgut, trunk kidney, head kidney, skin, muscle, gill, gonad, liver, spleen, and heart. Total RNA was extracted to generate the first cDNA template using a Hifair^®^ Ⅲ 1st Strand cDNA synthesis kit (Yeasen, Shanghai, China), according to the manufacturer’s instructions, for performing the quantitative real-time PCR (qPCR) experiments. The qPCR primers used in this experiment were designed based on the trans-intron principle, and the amplification conditions involved an initial denaturation step at 94 °C for 5 min, followed by 35 cycles of 30 s at 95 °C, 45 s at 60 °C, 80 s at 72 °C, and finally an extension step at 72 °C for 15 min. The primers were subsequently verified using 1.0% agarose gel electrophoresis and were considered good when the results showed only one bright target band with a length of 100–200 bp. Primer specificity was further validated via melting curve analysis after amplification during subsequent qPCR analyses. Elongation factor 1α (EF-1α) was used as the internal reference gene to normalize gene expression. The assay procedure was performed on Quant Studio TM Design & Analysis Software 3.0 on a real-time fluorescence quantitative PCR system combined with 2 × SYBR qPCR Mixture (HLINGENE, Shanghai, China) to detect the mRNA expression of IL-10 and IL-6 in different tissue samples; each sample was tested in triplicate. The qPCR reaction system contained 5 µL of 2 × SYBR qPCR mixture, 4.5 µL of the cDNA template, and 0.25 µL of each primer (10 µM) and reached a final volume of 10 µL. The qPCR procedures used were as follows: 94 °C for 2 min, 94 °C for 12 s, 60 °C for 15 s, and 72 °C for 25 s for a total of 40 cycles. The relative expression levels of all IL-10 and IL-6 were analyzed using the 2^−ΔΔCt^ method.

### 2.5. In Vitro CCK Culture and Immune Induction

Channel catfish kidney (CCK) cells were provided by the National Pathogen Collection Center for Aquatic Animals, Shanghai Ocean University. The cells had been preserved in liquid nitrogen and thawed in M199 medium containing 10% fetal bovine serum (FBS, Lonsera, Gibco, Thermo Fisher Scientific, America) and 1% penicillin and streptomycin (100 U/mL penicillin and 100 µg/mL streptomycin), which was incubated at 28 °C. After counting, the cells were added into four wells with 2 mL of the mixture (10^6^ cells/well) for 6 h. After they were wall-stabilized, the cells were incubated with LPS (50 µg/mL), polyinosinic-polycytidylic acid (Poly(I:C)) (50 µg/mL), phytohaemagg lutinin (PHA) (10 µg/mL), and phorbol ester (PMA) (0.5 µg/mL), which were added to stimulate the cells. LPS, Poly(I:C), PHA, and PMA were purchased from Shanghai Yuanye Bio-Technology Co., Ltd. (Shanghai, China). The control groups were treated with the same dose of sterile PBS, four replicates of each treatment were created, and the cells were collected 24 h and 48 h post-stimulation. All these cells were collected for RNA extraction.

### 2.6. Bacterial Challenge

*Yersinia ruckeri* (GenBank accession number is OL376599) [29] was reported in our previous study, and *Streptococcus iniae* was provided by Northwest A & F University. The bacteria were cultured on brain heart infusion (BHI) at 28 °C for 18 h to reach the logarithmic growth phase and were then rinsed with sterile phosphate buffer saline (PBS) and adjusted to a concentration of 1.5 × 10^7^ CFU/mL. In the bacterial challenge experiment, the experimental group was injected with 200 µL of the bacteria through intraperitoneal injection, while the other group was injected with an equal volume of PBS as the control. *S. iniae* injection experiments were performed with reference to *Y. ruckeri*. A total 360 fish were divided into four groups, which consisted of the experimental groups of *Y. ruckeri* and *S. iniae* and their corresponding control groups, with 90 fish in each group. The gill, skin, hindgut, spleen, trunk kidney, and head kidney tissues were collected from the control and experimental groups 0, 6, 12, 24, and 48 h after injection, with four parallel samples performed at each sampling time point. These samples were treated with TRIzol reagent, and total RNA was extracted and reverse transcribed into cDNA to be used for the expression analysis.

### 2.7. Viral Infection

The channel catfish virus (CCV) was provided by the Huazhong Agricultural University. In the infection experiment, 194 channel catfish were randomly divided into two groups, with 97 fish in each group. The fish were injected with 80 µL of the CCV solution (resuspended in DMEM, 1 × 10^7^ TCID_50_/mL) or the same DMEM. Four channel catfish were selected, and then the gill, skin, hindgut, spleen, trunk kidney, and head kidney tissues were sampled at 1 d, 3 d, and 5 d, and were homogenized using the TRIzol reagent for total RNA extraction and cDNA synthesis.

### 2.8. Deltamethrin Challenge of the Channel Catfish

Deltamethrin was purchased from Shanghai Yuanye Bio-Technology Co., Ltd. For the deltamethrin immersion experiment, the 300 fish were divided into low-concentration and high-concentration groups, with the same number of fish in each group. After soaking the fish in either a low concentration (0.5 µg/L) or high concentration (5 µg/L) of deltamethrin, the six tissues were collected, and four parallel samples were performed at each sampling time point. After homogenization using TRIzol reagent, total RNA was further extracted, and cDNA was synthesized to analyze gene expression using qPCR experiments.

### 2.9. Statistical Analysis

All data were expressed as mean ± standard error of the mean (SEM). Statistical analysis was performed using SPSS 25.0 software. One-way ANOVA and t-test were used to determine the significance (* *p* < 0.05 or ** *p* < 0.01) between the treatment group and the control group.

## 3. Results

### 3.1. Sequence Analysis of the IL-10 and IL-6 Genes in Channel Catfish

**Figure 1:**
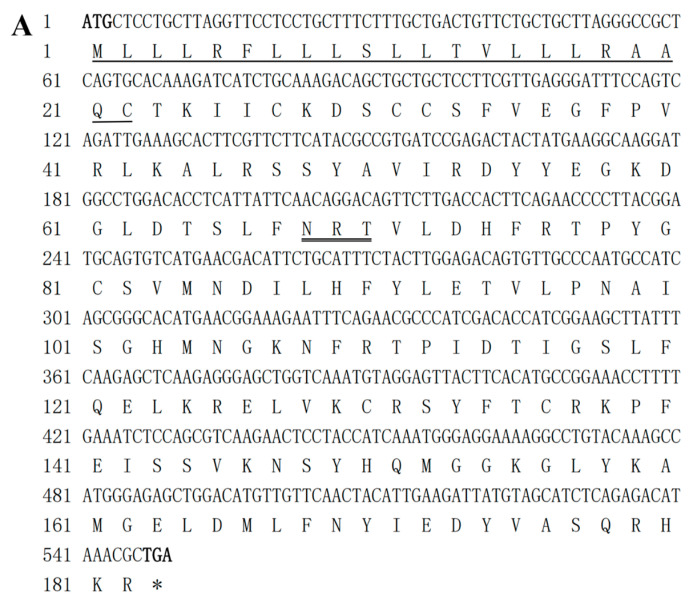

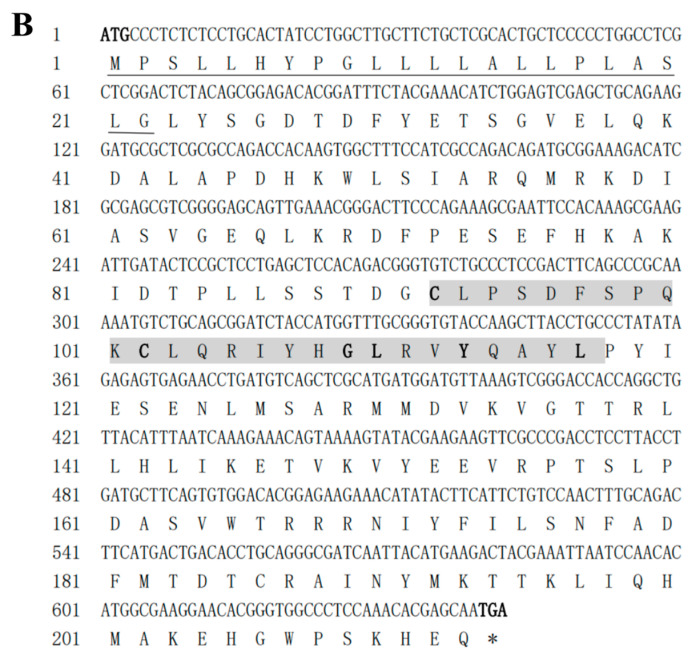
The CDS sequence and predicted amino acid sequences of IL-10 (**A**) and IL-6 (**B**). The predicted signaling peptides are underlined, and the start and stop codons are in bold. Asterisks (*) are used to indicate stop codons. The predicted N-glycosylation sites are double-underlined. The IL-6 family signature (C-X(9)-C-X(6)-G-L-X(2)-Y-X(3)-L) is shaded.

A 549 bp CDS region of IL-10 was obtained (GenBank number: OM641813.1) (Figure 1A), and it was found to encode for 182 amino acids with an isoelectric point of 9.17 and a molecular mass of 20.99 KDa. It contained an N-terminal glycosylation site (N-X-(S/T), X for any amino acid). The channel catfish IL-10 protein sequence showed the highest level of homology with IL-10 homologs from teleost fish. IL-10 is a helix-rich cytokine consisting of six alpha helices.

The sequence of the IL-6 CDS region (GenBank number: OM641812.1) obtained was 642 bp long, and the signaling peptide consisted of amino acid residues 1–22, which were predicted to encode for 213 amino acids, with an isoelectric point of 7.75 and a molecular mass of 24.48 KDa. This IL-6 sequence (C-X(9)-C-X(6)-G-L-X(2)-Y-X(3)-L) was a consensus marker of the IL-6 gene in channel catfish (Figure 1B). The homology analysis showed that the IL-6 gene of the channel catfish was most closely related to the IL-6 gene of *A. melas* and contained four α-helix structures.

### 3.2. Phylogenetic Analysis and Multiple Alignment

**Figure 2:**
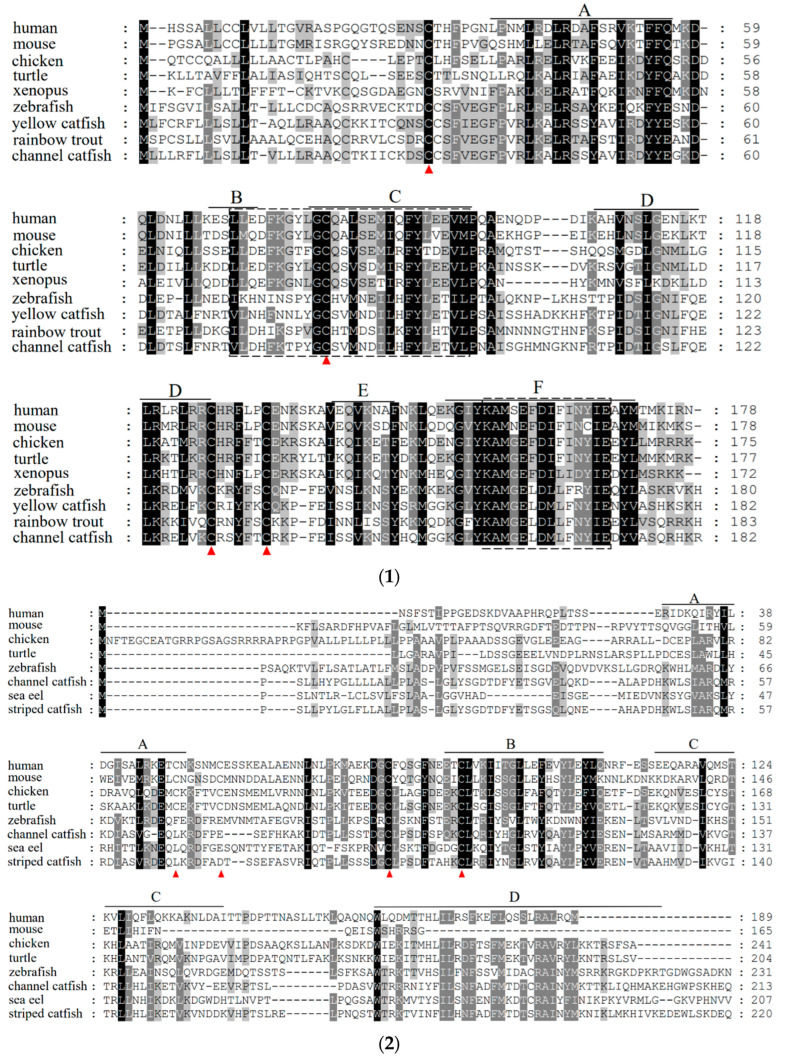
Alignment of the deduced amino acid sequence of the channel catfish IL-10 gene (**1**) and IL-6 gene (**2**). The position of A—F α-helix are underlined. The four conserved cysteine residues are indicated using red arrows. The identical residues are colored in black (75.0–99.9% aa sequence identities), while other, partly conserved residues are colored in dark grey (50.0–74.9% aa sequence identities).

**Figure 3:**
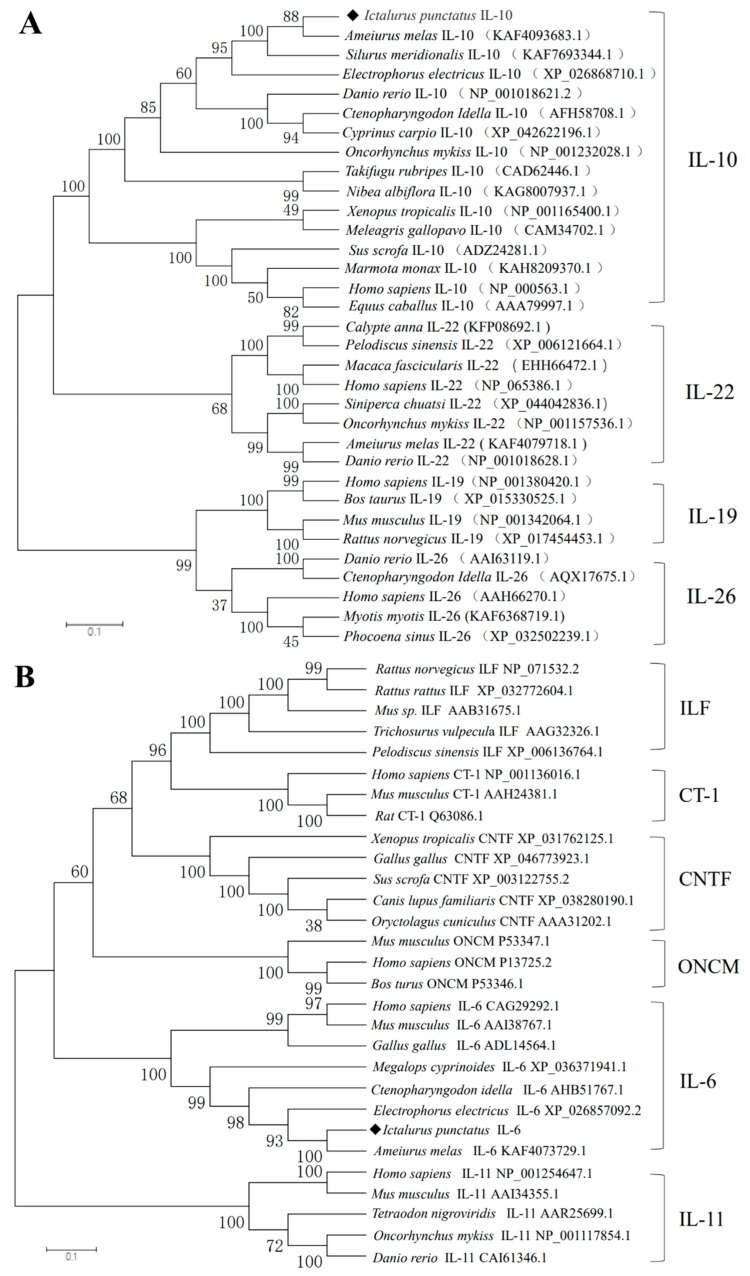
Phylogenetic analysis of the IL-10 family (**A**) and the IL-6 family (**B**) of cytokines. The phylogenetic trees were constructed using the neighbor-joining (NJ) method supported by 1000 bootstrap replications performed in MEGA 6.0 software. The channel catfish are indicated by the sign ◆.

**Figure 4:**
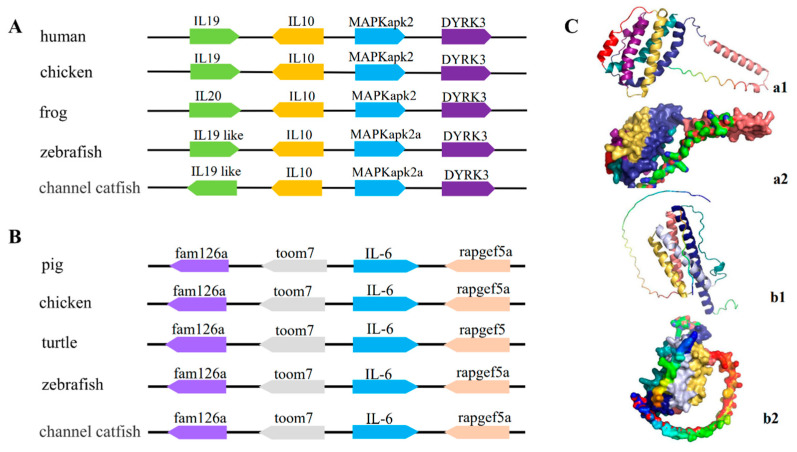
Gene synteny analysis of the IL-10 gene (**A**) and the IL-6 gene (**B**). Arrow direction indicates gene transcription orientation. The predicted three-dimensional structures of the channel catfish IL-10 gene ((**C**) a1and a2) and IL-6 gene ((**C**) b1 and b2).

The IL-10 gene contained four conserved cysteine residues in all sequences compared, forming two disulfide bonds in the same manner. The channel catfish IL-10 gene contained the IL-10 family signature motifs (LDHFRTPYGCSVMNDILHFYLETVL) and (KAMGELDMLFNYIE) (Figure 2-1). The IL-6 gene of channel catfish, just like that of other fish, has only two conserved cysteine residues out of the four involved in disulfide bond formation (Figure 2-2). To elucidate the evolutionary relationships between IL-10, IL-6, and other family members, we constructed the respective phylogenetic trees. The phylogenetic tree of IL-10 contains the IL-10, IL-22, IL-19, and IL-26 of the IL-10 family (Figure 3A). The phylogenetic tree of IL-6 contains ILF, CT-1, CNTF, ONCM, IL-6, and IL-11 of the IL-6 family (Figure 3B). Each gene mainly had a mammalian, amphibian, and fish version. The overall analysis showed that, evolutionarily, the closest affinity between channel catfish and teleost fish were located in the IL-10 and IL-6 branches, respectively. The gene synteny analysis showed that the IL-10 and IL-6 genes are highly conserved during evolution. Many conserved genes, for example, MAPKapk2 and DYRK3, which are highly linked with the IL-10 locus, were found in all the analyzed species (Figure 4A). IL-6 genes are also highly linked to conserved genes, such as fam126a, toom7, and rapgef5a/rapgef5 (Figure 4B). We observed the tertiary structure of the IL-10 and IL-6 genes in channel catfish (Figure 4C); the predicted 3D structures of IL-10 and IL-6 protein mainly constitute alpha helices and random coils.

### 3.3. Tissue Expression Analysis of IL-10 and IL-6

**Figure 5:**
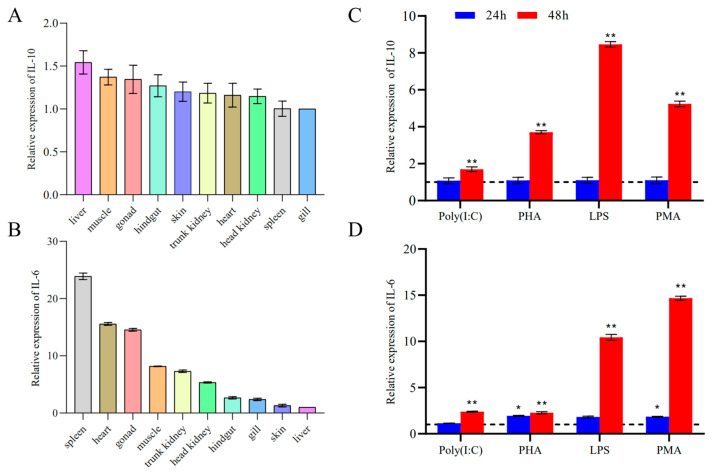
Tissue distribution of IL-10 (**A**) and IL-6 (**B**) in healthy channel catfish. Among the tissues examined, the lowest level of IL-10 and IL-6 was expressed in the gill and liver, respectively, and this was chosen for calibration, while the relative expression levels of IL-10 and IL-6 in the other tissues are presented as fold-changes based on the calibration. The data are expressed as mean ± SEM (*n* = 4) and were normalized to the level of EF-1α expression in the same samples. Expression analysis of IL-10 (**C**) and IL-6 (**D**) expression, as shown by the CCK assay. The cells were stimulated with Poly (I:C), PHA, LPS, or PMA for 24 h and 48 h and were analyzed by performing qPCR. The data are expressed as mean ± SEM (*n* = 4). Significant differences are indicated using asterisks (* *p* < 0.05, ** *p* < 0.01).

Both IL-10 and IL-6 were detected in the hindgut, trunk kidney, head kidney, skin, muscle, gill, gonad, liver, spleen, and heart of healthy fish. The tissue distribution assays showed that channel catfish mRNA was expressed in all selected tissues. In healthy channel catfish, IL-10 was expressed in the liver, muscle, gonad, hindgut, skin, trunk kidney, heart, head kidney, spleen, and gill (Figure 5A). The expression levels of IL-6 varied considerably among the tissues examined, with the spleen displaying the highest level of expression (Figure 5B).

### 3.4. Modulation of IL-10 and IL-6 Expression in Channel Catfish Kidney (CCK) Cells

There was no effect on the LPS, Poly(I:C), PHA, and PMA expression levels of IL-10 and IL-6 at 24 h, while a stimulatory effect on CCK was observed after 48 h, as shown by the significant increase in the expression levels of IL-10 (Figure 5C) and IL-6 (Figure 5D).

### 3.5. Analysis of IL-10 and IL-6 Expression in Fish

**Figure 6:**
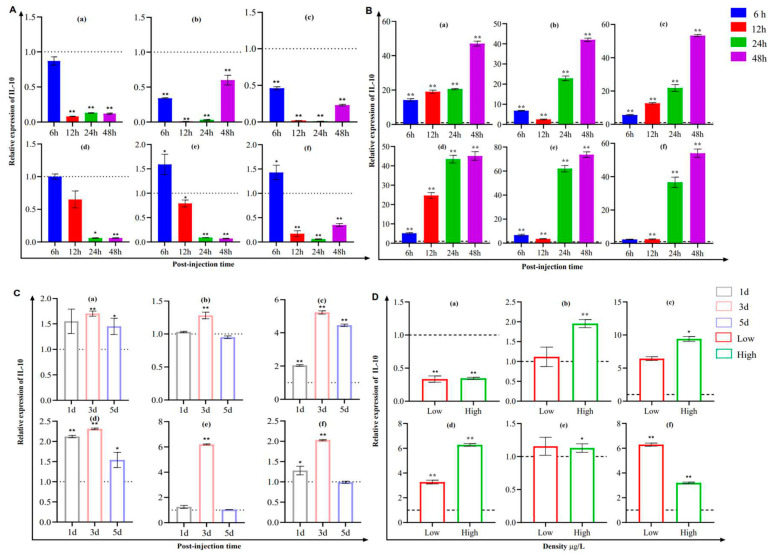
The mRNA expression analysis of IL-10 expression in the gill (a), skin (b), hindgut (c), spleen (d), trunk kidney (e), and head kidney (f) of channel catfish after being challenged by *Y. ruckeri, S. iniae*, CCV, and deltamethrin, as shown in (**A**–**D**), respectively (*n* = 4). Significant differences are indicated using asterisks (* *p* < 0.05, ** *p* < 0.01).

The expression of IL-10 significantly increased in the trunk kidney and head kidney after *Y. ruckeri* was challenged for 6 h (Figure 6A). We observed a clear time-dependent trend in the expression of IL-10 in the six types of tissues after *S. iniae* stimulation, which reached peak levels at 48 h (Figure 6B). Moreover, the maximum level of expression was reached on the third day in the presence of CCV (Figure 6C), with the expression of IL-10 being 4.2 and 2.13 times higher than that of the unstimulated state. Similarly, the expression levels were significantly upregulated in the skin, hindgut, trunk kidney, and head kidney at low and high levels of deltamethrin concentration, and the difference in the change in expression was highly significant at a high level of deltamethrin (Figure 6D).

**Figure 7:**
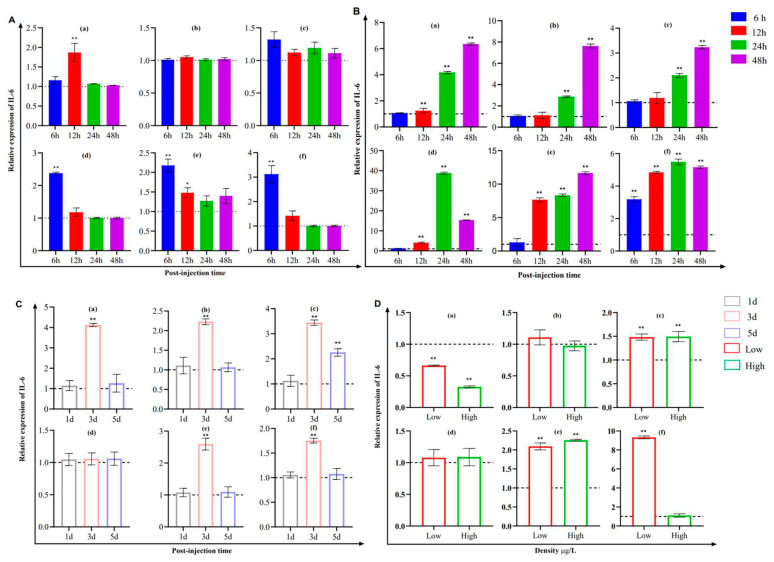
The mRNA expression analysis of IL-6 expression in the gill (a), skin (b), hindgut (c), spleen (d), trunk kidney (e), and head kidney (f) of channel catfish after being challenged by *Y. ruckeri*, *S. iniae*, CCV, and deltamethrin, as shown in (**A**–**D**), respectively (*n* = 4). Significant differences are indicated using asterisks (* *p* < 0.05, ** *p* < 0.01).

The expression of IL-6 in the gill, spleen, trunk kidney, and head kidney showed an acute phase response at 6 h after *Y. ruckeri* stimulation (Figure 7A), similar to the expression levels of IL-10 and IL-6, which increased gradually after *S. iniae* stimulation (Figure 7B). On the third day, the expression levels were elevated and then returned to normal levels after CCV stimulation (Figure 7C). Under the effect of deltamethrin, the expression level of IL-6 showed a decreasing trend in the gill but was significantly and highly elevated in the hindgut, trunk kidney, and head kidney (Figure 7D).

## 4. Discussion

In this study, we reported on the molecular cloning and gene expression analysis of the IL-10 and IL-6 genes in channel catfish. Both genes were found to be structurally similar to *A. melas,* the predicted signaling peptide, as indicated by the secreted protein. Similar to that of the other fish, the sequences of the channel catfish IL-10 gene contained six alpha helixes with four cysteine residues that formed two disulfide bonds in the same manner as that of mammalian and human IL-10 [30], which is essential for the maintenance of structure and biological activity. Among them, the alpha helixes A, B, and F act as potential receptor-binding domains and are often activated by forming receptor-bound dimeric structures [31]. Previous mammalian studies have shown that the four conserved cysteine residues in IL-6 are involved in the formation of two disulfide bonds [18], and multiple sequence comparisons have shown that the first two cysteines, which are found in mammals, are absent in fish, suggesting that the biological activity of that disulfide bond may not be mandatory in fish. The phylogenetic tree analysis of our sequences alongside other IL-10 and IL-6 family members showed that channel catfish IL-10 and IL-6 cluster together with other teleost isoforms, which is supported by 100% bootstrap values. This indicated the stable and conserved nature of the structures of IL-10 and IL-6 in channel catfish.

The expression analysis of both genes showed that IL-10 and IL-6 were expressed in all tissues tested, which is consistent with the findings of other fish using qPCR methods. The tissue expression pattern of the interleukin gene family showed variability in fish due to fish species, although they are closely related phylogenetically. The high expression of the channel catfish IL-10 gene in liver tissue is in agreement with the findings in the spotted knifejaw (*Oplegnathus punctatus*) [32]. IL-10 expression was most elevated in the spleen tissue of yellow catfish [33]. In addition, nested PCR detected very low levels of IL-10 expression in the gills and gonads of healthy pufferfish [34]. These results seem to confirm the association of IL-10 with the immune system of fish species [5]. Consistent with previous findings on blunt snout bream (*Megalobrama amblycephala*) [35] and Siberian sturgeon (*Acipenser baeri*) [21], the basal conditions showed the highest level of IL-6 expression in the spleen, an important tissue that mediates the immune response, suggesting that the response of IL-6 in immune organs highlights its key role in the immune system.

IL-10 plays an important role in the inflammatory response of the body as well as in tissue damage [36]. Previous reports have shown that both IL-10 and IL-6 expression can activate the JAK/STAT3 signaling pathway and play a key role in immunomodulatory processes [37,38]. In addition, IL-10 mediate the immune responses of bacterial infections, as demonstrated in fish and exemplified in sea bass (*Dicentrarchus labrax L.*) [39] and Indian major carp (*Catla catla*) [6], in which bacteria successfully induced IL-10 expression in the kidney (an immune organ). In addition to the stimulation of golden pompano by *Streptococcus agalactiae* [5] and infection of spleen and kidney necrosis virus (ISKNV) in mandarin fish (*Siniperca chuatsi*) [40], IL-10 expression was significantly upregulated in the spleen after stimulation. This is consistent with the conclusion that IL-10 is associated with the innate immune system in mammals [41]. A similar trend in IL-10 expression was observed in the gill, skin, hindgut, spleen, trunk kidney, and head kidney tissues of the channel catfish in the present investigation after stimulation by bacteria and the channel catfish virus. These responses provided ample evidence that IL-10 is a key immunomodulatory cytokine during infection and is also required for optimal pathogen clearance and ameliorates immunopathological status [42].

IL-10 can attenuate *Mycobacterium avium* and *Klebsiella pneumonia* infection in mammalian mice [43,44]. In fish, the results showed that IL-10 could indirectly weaken immune resistance to *Mycobacterium marinum* infection in zebrafish [45]. In this study, IL-10 expression after stimulation using the Gram-negative bacteria, *Y. ruckeri*, showed a decrease in expression in all fish tissues analyzed. This is consistent with the findings observed in previous studies [40,45].

IL-6 has been shown to play an important role in the host response to many bacterial and viral infections [20,46,47]. For example, the intestine, spleen, and liver of *Megalobrama amblycephala* were attacked by *Aeromonas hydrophila* [35], and IL-6 expression was found to be significantly elevated in rainbow trout mononuclear phagocytes [19] after LPS stimulation when compared with the controls. Our experiments showed the time-dependent and tissue-specific expression of upregulation after bacterial attack. *S. iniae*-infected channel catfish continued to show the upregulated expression of IL-10 6–48 h after infection, with a peak at 48 h. This suggests that IL-6 is involved in defense mechanisms against bacteria through its elevated expression, induced by a bacterial attack, along with the transient responses of *Y. ruckeri* at 6 h in the trunk kidney and head kidney after the host was attacked, where the early induction of the expression of IL-6 was associated with acute-phase proteins that affect cellular transport and mediate release to enhance protection against micro-organisms [35]. The transient response of IL-6 expression may be due to the exposure of immune cells to bacteria, as observed by the associated molecular patterns of the pathogen-associated molecular patterns (PAMPs) under pathogen attack [24], and the intense attack response of fish immune cells and tissues to pathogens in vivo, which may suggest that the heterogeneous populations of certain immune cells may secrete pro-inflammatory cytokines that rapidly upregulate IL-6 mRNA expression levels within a short period of time.

Low-residue deltamethrin disrupts the balance of the aquatic ecosystem when used in large quantities in aquaculture. Due to the lipophilic nature of deltamethrin [48], it can easily enter the fish body through the gill and subsequently produce strong toxic effects in the gill and liver of zebrafish through blood circulation [49]. After deltamethrin immersion, IL-10 and IL-6 expression in the skin, hindgut, trunk kidney, and head kidney were upregulated in the channel catfish, and the opposite trend was observed in the gills. When the channel catfish were exposed to appropriate concentrations of deltamethrin, the increase in free amino acid levels in response promoted energy availability [50]. Therefore, it is also likely to exert an activating effect on the immune system. In addition, this could also be a result of the damage to immune cells, especially mucosal tissues, caused by high levels of stress, which inhibits their expression. The present study further verified that deltamethrin can cause lesions in the gill epithelium of fish [51,52]. Additionally, the hindgut plays a key role in intestinal immunity, and the toxic effects of deltamethrin at 5 µg/mL exerted for a short period of time can induce changes in the intestinal flora, the shedding of intestinal villous epithelial cells, and the activation of the associated immune response [53]. The upregulation of IL-10 and IL-6 expression in the hindgut was also an auto-protective behavior, as shown in our experiment. This also shows that deltamethrin, as a representative pyrethroid insecticide, should be used carefully on aquatic animals.

In summary, we identified homologs of IL-10 and IL-6 in channel catfish and found that different pathogenic micro-organisms could successfully induce the expression levels of IL-10 and IL-6 in vivo and in vitro in channel catfish. This emphasized that the involvement of IL-10 and IL-6 in the immune response of channel catfish plays a key role in assisting with organism protection against pathogenic infections as well as tissue repair. We hope our findings will provide new insights into the function of fish IL-10 in host immune infections.

## Figures and Tables

**Table 1:** Primers used in this study.

Gene	Primer Sequence (5′-3′)	Application
IL-10-F	ATGGGAAGGAATTTTTGGGC	PCR
IL-10-R	TCAGCGTTTATGTCTCTGAG	PCR
IL-6-F	ATGCCCTCTCTCCTGCACTATCCTG	PCR
IL-6-R	TCATTGCTCGTGTTTGGAGGGCCAC	PCR
EF-1α-F	GTTGAAATGGTTCCTGGCAA	qPCR
EF-1α-R	TCAACACTCTTGATGACACCAAC	qPCR
IL-10-F	GCTTAGGGCCGCTCAGTGCA	qPCR
IL-10-R	GCCTTCATAGTAGTCTCGG	qPCR
IL-6-F	CAGCCCGCAAAAATGTCTGC	qPCR
IL-6-R	TCAGGTAAGGAGGTCGGGCG	qPCR

## Data Availability

Data available on request from the authors.
